# Poor quality sleep is associated with greater carotid intima media thickness among otherwise healthy resident doctors

**DOI:** 10.3389/fepid.2022.1044111

**Published:** 2023-01-11

**Authors:** Prativa Priyadarshani Sethi, Monika Pathania, Ravi Gupta, Pankaj Sharma, Lokesh Kumar Saini

**Affiliations:** ^1^ Department of Internal Medicine, All India Institute of Medical Science, Rishikesh, India; ^2^ Department of Psychiatry, All India Institute of Medical Science, Rishikesh, India; ^3^ Department of Radiodiagnosis, All India Institute of Medical Science, Rishikesh, India; ^4^ Department of Pulmonary Medicine, All India Institute of Medical Science, Rishikesh, India

**Keywords:** physician health, subclinical atherosclerosis, sleep hygiene, sleep medicine, cardiovascular medicine

## Abstract

**Background:**

Sleep is important for maintaining the metabolic processes in the body, and hence, disruption in sleep leads to metabolic derangement and accelerated atherosclerosis. The effect of sleep duration on subclinical atherosclerosis has been examined in several studies; however, data regarding sleep quality is lacking. The study aimed to assess the association between sleep quality and carotid intima-media thickness among healthy young doctors.

**Materials and Methods:**

This was an observational cross-sectional study among 110 healthy young resident doctors. Anthropometric data were recorded and morning fasting venous blood samples were collected to assess fasting blood sugar, lipid profile and glycosylated haemoglobin (HbA_1c_). Pittsburgh Sleep Quality Index and Berlin questionnaire assessed subjective sleep quality and risk for obstructive sleep apnea, respectively. Carotid ultrasonography was done to detect the intima-media thickness.

**Results:**

Average age of the participants was 26.45 (±1.43) years, and 51.8% were male. Self-reported poor sleep quality was found in 54.5%. Carotid intima-media thickness (CIMT) was increased among 44.5% of participants. In the multivariate analysis, only poor sleep quality appeared to be associated with higher CIMT (*P* < 0.001, OR = 7.4; 95% CI = 2.70–20.32). When different components of sleep quality was analyzed through multivariate logistic regression, subjective sleep onset latency (>30 min), sleep efficiency (<85%) and sleep disturbance was found to be associated with the increased CIMT.

**Conclusion:**

Poor sleep quality, especially prolonged sleep onset latency, poor sleep efficiency, and sleep disturbance are associated with increased carotid intima-media thickness among healthy young adults.

## Introduction

Medical residents spend long hours in patient care as well as study, resulting in sleep deprivation (1). A decent amount of literature has discussed sleep deprivation among medical residents and its adverse consequences on social, health and occupational aspects (1). Sleep deprivation has been found to impair cognition, prolong reaction time, enhance errors, sleepiness, irritability, reduce empathy, motor vehicle accidents, and increase the use of hypnotics and alcohol (1). Optimal sleep duration is important as both long (>9 h/day) as well as short (<7 h/day) have been found to increase the risk for cardiovascular disorders, including systemic hypertension, diabetes mellitus, metabolic syndrome and mortality from cardiovascular disorders (2). Both long and short sleep duration has been found to increase the carotid intima-media thickness (CIMT), however recent literature gives a contrasting evidence showing no association between sleep duration and CIMT (3–6).

Sleep duration and sleep quality represent two different aspects of sleep-related health (7). Optimal duration of sleep for an individual has been conceptualized as one which provides a feeling of “rested” upon awakening and allow one to perform well during the day with a considerable inter-individual variation (8). Considering this, it may be possible that reported short or long sleep duration in previous studies was physiological, at least for a few participants (3–5). Hence, assessing sleep duration without considering sleep quality may introduce bias in the studies correlating CIMT with sleep duration (3–5, 7).

While sleep duration is determined by an interplay of genetic, environmental and health-related factors, including the use of exogenous chemicals, viz., addictive substances and medications, sleep quality is a broader concept (8). Determinants of sleep quality among young adults include physical activity, quality of social interactions, stimulants intake, stress, sleep-wake patterns, sleep onset latency, wake after sleep onset, number of awakenings and total sleep time (9, 10). Sleep quality is also affected by sleep disorders like obstructive sleep apnea, insomnia and restless legs syndrome (11–13). Hence, sleep quality provides a comprehensive assessment of sleep and daytime functions compared to sleep duration. Sleep quality is a subjective phenomenon, and literature suggests that actigraphy and polysomnography data has a poor correlation with subjectively reported sleep quality (10, 14).

Carotid intima media thickness (CIMT) is a reliable marker of early atherosclerosis and hence a predictor for atherosclerotic cardiovascular disease (15, 16). The evidence till date have been derived from middle to old age population with comorbidities (5, 15, 17–19) except a few study which is done among young adult (20, 21). Increasing age has been a consistent independent risk factor for increased CIMT (18, 21, 22).

Literature regarding the assessment of sleep quality among medical residents is limited (23–26). Available studies show that 45% to 90% of medical residents report poor sleep quality. Poor sleep quality has been associated with fatigue, memory lapses, perceived stress, heartburn, irritable bowel syndrome and depression among medical residents (23–25). Considering the inclusive nature of “sleep quality”, it is possible that sleep quality might influence the CIMT among medical residents, as has been observed among middle-aged women (27). However, such data is not available. We hypothesized that poor sleep quality is associated with greater CIMT in medical residents, an otherwise healthy young population, similar to what has been reported among middle-aged women even after excluding potential confounders.

## Materials and methods

The study was a cross-sectional descriptive study conducted among apparently healthy resident doctors in a teaching hospital. The study was conducted after getting approval from the Institutional Ethics committee (Letter number-****/IEC/20/857). Based on the previous literature, the sample size was calculated using the prevalence of sleep disturbance (7%) among resident doctors of a teaching institute (28). Considering 95% confidence interval and 5% absolute precision, the estimated sample size was 100. 10% of participants were added to address non-response.

The study was planned in a teaching hospital to recruit healthy young participants. The postgraduate residents from all the departments of same year accounting to 121, were listed. It helped minimising heterogeneity among study participants in terms of duty hours, shift duty, and level of stress. All the residents were below 30 years.. After the “line listing” of all resident doctors meeting the age criteria, participant selection was made using a “simple random sampling” method [sequence generated by open access computer-based software programme (*QuickCalcs* http://www.graphpad.com/quickcalcs/)].

Eligible study participants were approached and explained about the rationale of the study. Written informed consent was obtained from those agreeing to participate. However, those with a known history of diabetes, hypertension, coronary artery disease and cerebrovascular disease were excluded. A family history of cardiovascular disease was also obtained from the participants, and those having a positive family history were considered to have an increased risk of atherosclerosis.

### Demography and anthropometric assessment

Demographic details included age, gender and marital status. Participants were individually assessed for their anthropometric measurements. Weight (Kg), height(cm), waist circumference and waist-hip ratio were measured as per standard guidelines (29). Body mass index (BMI) was calculated, and groups were made as per guidelines for the Asian population (30). Blood pressure measurement was done using an aneroid sphygmomanometer following the auscultatory method for each participant. The measurement was taken twice, and the average was recorded. Participants having systolic blood pressure(SBP) ≥ 140 mmHg or diastolic blood pressure(DBP) ≥ 90 mmHg on two occasions a few days apart were considered to have hypertension (31).

### Assessment of sleep quality

Subjective sleep quality was assessed using Pittsburgh Sleep Quality Index (PSQI) (32). It differentiates “poor” from “good” sleep quality based on a global score which is calculated using seven components- subjective sleep quality, sleep latency, sleep duration, habitual sleep efficiency, sleep disturbances, use of sleeping medications, and daytime dysfunction over the last month. Global PSQI score ≤5 indicates good sleep quality, while a score of >5 shows poor sleep quality. Using a cut-off score of 5, PSQI has a sensitivity of 89.6% and specificity of 86.5%, respectively, to differentiate between good and poor sleep quality (32).

### Risk for obstructive sleep apnea (OSA)

OSA risk was assessed using the Berlin questionnaire(BQ) as the sleep laboratory was closed during the Corona virus disease-19 (COVID-19) pandemic (33). It consists of 3 categories which ultimately classifies the participants into low risk or high risk of sleep apnea. BQ has 86% sensitivity and 77% specificity to differentiate between how and high risk of OSA. Its positive predictive value is 0.89 (33). A meta-analysis has shown that it is a good screening tool for OSA (34).

### Other variables

Metabolic syndrome was diagnosed based on the National Cholesterol Education Program and Adult Treatment Panel III (NCEP-ATP III) criteria (35). Questions were asked about the present status of smoking, and participants were classified into two categories-smoker and non-smoker). Similarly, based on usual dietary habits, participants were categorized into vegetarian and non-vegetarian.

### Carotid artery intima-media thickness

Carotid intima-media thickness (CIMT) was assessed by high-resolution B-mode ultrasonography (SonoSite machine- M-Turbo; Fujifilm SonoSite Inc., USA) with a broadband width linear array transducer (6–13 MHz probe) following standard guidelines (36). The radiologist performing CIMT measurement was kept blinded to participants' sleep and other health-related data to remove the bias. Measurements were taken in the supine position with the head of the participant resting comfortably, the neck slightly hyper-extended and rotated opposite the probe. A wedge pillow at an angle of 45° was used to ensure lateral rotation. On longitudinal 2D ultrasound images of the carotid artery, the near and far walls were displayed as two echogenic lines (the adventitia and intima), separated by the hypoechoic media. The distance between the first bright line of the far wall (lumen-intima interface) and the second bright line (media-adventitia interface) was defined as the CIMT. This study measured right and left side IMT distal 10 mm of the common carotid artery in the far wall. Six values of CIMT (three on each side) were obtained, and the average of these measurements was taken for final analysis. Carotid IMT >0.7 mm was considered as increased, and ≤0.7 mm was taken as normal (37).

### Laboratory investigations

Lipid profile was measured after eight hours of fasting (Beckman coulter AU680 machine; Beckman Coulter, Inc., USA and EMBEE diagnostic reagent kit; Embee Diagnostics, India).

Fasting blood sugar (FBS) was measured for each participant after 8 h of fasting by the hexokinase method. (Beckman coulter AU480 machine Beckman Coulter, Inc., USA and EMBEE diagnostic reagent kit; Embee Diagnostics, India).

Glycated haemoglobin (HbA_1c_) was assessed for each participant using the high-performance liquid chromatography method (G8 90SL model, Tosoh Bioscience, Inc., USA).

## Statistical analysis

The data was analyzed using Statistical Package for Social Sciences (SPSS) version 23.0 (IBM Corp. Released 2015. IBM SPSS Statistics for Windows, Version 23.0. Armonk, NY). The Shapiro-Wilk test was applied to check the normality of data. Descriptive statistics were calculated. Statistical significance of qualitative variables was calculated using the Chi-Square test. Quantitative variables having normal distribution were compared using an independent sample t-test. Variables having statistical significance in univariate analysis were considered for multivariate logistic regression analysis to develop a model for increased CIMT.

## Results

**Figure 1:**
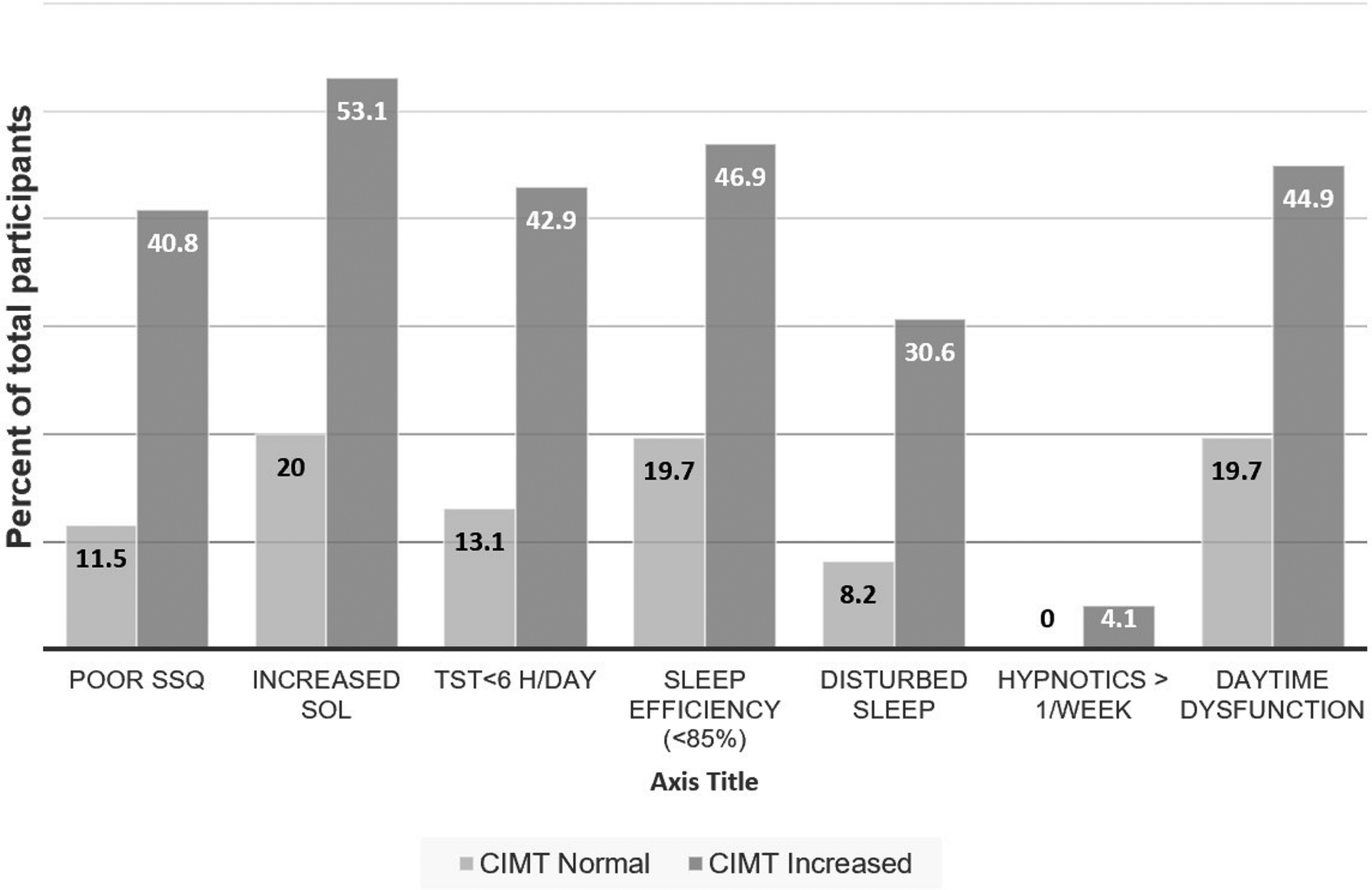
Proportions of subjects having dysfunction on various components of Sleep Quality. SSQ-subjective sleep quality, SOL-sleep onset latency, TST-total sleep time.

A total of 110 participants were enrolled, and the response rate was 100%. The average age was 26.45 (±1.43) years with approximately equal gender distribution (51.8% male). 57.3% had normal BMI, 36.4% were obese grade 1, and 6.3% were obese grade II according to Asian-Pacific cut off points (30). There was no racial and ethnicity difference among the participants. None of the participants had family history of coronary and cerebrovascular disease. None of the participants reported sleeping pill usage. Carotid IMT was increased (>0.7 mm) among 44.5% of participants. Comparison of clinico-demographic factors between participants with normal and increased CIMT is depicted in Table 1. Poor sleep each of the components of the PSQI questionnaire was associated with increased CIMT (Figure 1).

**Table 1:** Comparison of factors associated with carotid intima-media thickness.

Variables	CIMT		*P*-value
	≤0.7 mm (*N* = 61)	>0.7 mm (*N* = 49)	
Male Gender	29(47.5%)	28 (57.1%)	0.31
Non-vegetarian food habit	38 (62.3%)	37 (75.5%)	0.13
History of smoking	18 (29.5%)	23 (46.9%)	0.06
Family history of coronary artery disease	19 (31.1%)	22 (44.9%)	0.13
Poor sleep quality	19 (31.1%)	41 (83.7%)	<0.001
Presence of Metabolic syndrome	10 (16.4%)	23 (46.9%)	0.001
BMI (>25 Kg/m^2^)	21(34.4%)	26(53.1%)	0.05
Triglyceride (>150 mg/dl)	15 (24.6%)	29 (59.2%)	<0.001
Total cholesterol(>200 mg/dl)	3 (4.9%)	9 (18.4%)	0.02
Low density lipoprotein(≥100 mg/dl)	19 (31.1%)	21 (42.9%)	0.20
Waist circumference (Male (>102 cm, Female(>88 cm)	8(13.1%)	17 (34.7%)	0.007
FBS (≥100 mg/dl)	6 (9.8%)	17 (34.7%)	0.001
High risk sleep apnea	1 (1.6%)	6 (12.2%)	0.04
	Mean ± SD		
Waist-Hip ratio	0.87 ± 0.08	0.91 ± 0.16	0.15
Age (Yrs)	26.36 ± 1.37	26.55 ± 1.5	0.49
Systolic blood pressure (mmhg)	114.03 ± 9.90	113.9 ± 10.21	0.97
Diastolic blood pressure (mmhg)	71.77 ± 8.44	72.98 ± 8.59	0.46
HbA1c	4.93 ± 0.42	5.16 ± 0.39	0.006

Comparison of Demographic and Laboratory factors between normal and increased CIMT. Categorical data are reported as counts with percent of the total sample in parenthesis, numerical data are reported as mean values ± standard deviation. BMI, body mass index; CIMT, carotid intima media thickness; FBS, fasting blood sugar; HbA1c, glycosylated hemoglobin; SD, standard deviation.

Multivariate logistic regression included poor sleep quality (as measured by global PSQI score) as well as other known risk factors for subclinical atherosclerosis. This model was overall significant [*χ*2 (9) = 43.57; *P* < 0.001], classified 75.4% cases correctly and showed 43.8% variation in the dependent variable (increased CIMT). Only poor sleep quality was associated with higher CIMT (*P* < 0.001, OR = 7.4; 95% CI = 2.70–20.32) (Table 2).

**Table 2:** Multivariate logistic regression model showing risk factors for increased carotid intima media thickness.

Variables	B	Standard Error	Wald	df	*P*	Odds Ratio	95% C.I for Odds Ratio	
							Lower	Upper
High risk for obstructive Sleep Apnea	−1.33	1.34	0.98	1	0.32	0.26	0.01	3.66
Poor Sleep Quality	2.00	0.51	15.19	1	<0.001	7.41	2.70	20.32
Presence of Metabolic Syndrome	0.10	1.01	0.01	1	0.91	1.11	0.15	8.17
Triglyceride (>150 mg/dl)	0.38	0.76	0.25	1	0.61	1.46	0.32	6.56
Total cholesterol (>200 mg/dl)	0.58	0.86	0.46	1	0.49	1.80	0.32	9.85
Waist circumference (≥102 cm in males and ≥88 cm in females)	0.006	0.95	<0.001	1	0.99	1.00	0.15	6.54
BMI (>25 kg/m2)	−0.02	0.53	0.002	1	0.96	0.97	0.34	2.77
FBS((≥100 mg%)	0.79	0.645	1.51	1	0.21	2.21	0.62	7.82
HbA1c (%)	0.30	0.64	0.22	1	0.63	1.35	0.38	4.82

BMI: body mass index, FBS: fasting blood sugar, HbA1c: glycosylated hemoglobin.

When the effect of different components of sleep quality on CIMT was analyzed through multivariate logistic regression, the model was overall significant [*χ*2 (7) = 39.70; *P* < 0.001], it classified 75.2% cases correctly and showed 40.8% variation in the dependent variable (increased CIMT). Subjective sleep onset latency, sleep efficiency and sleep disturbance was associated with higher odds for increased CIMT (Table 3).

**Table 3:** Multivariate logistic regression model of sleep components of PSQI for increased carotid intima media thickness.

Components of PSQI	B	Standard Error	Wald	df	*P*	Odds Ratio	95%C/I for Odds Ratio	
							Lower	Upper
Sleep Latency	1.42	0.51	7.56	1	0.006	4.14	1.50	11.430
Sleep duration	0.947	0.63	2.24	1	0.13	2.57	0.74	8.89
Sleep Efficiency	1.12	0.55	4.13	1	0.04	3.08	1.04	9.15
Sleep disturbance	1.35	0.67	4.06	1	0.04	3.89	1.03	14.56
Daytime dysfunction	0.78	0.52	2.25	1	0.13	2.20	0.78	6.15
Subjective Sleep Quality	1.086	0.65	2.76	1	0.09	2.96	0.82	10.65

PSQI, pittsburgh sleep quality index.

## Discussion

The present study involving 110 young, apparently healthy adult post-graduate medical residents showed that 54.5% of the participants reported their sleep quality as poor, and 44.5% had increased CIMT. Multivariate analysis depicted that after controlling for all confounders, only subjective poor sleep quality was associated with increased odds for increased CIMT (Table 2), especially sleep onset latency > 30 min, sleep efficiency <85% and sleep disturbance (Table 3).

This study showed that nearly half of the randomly selected resident doctors reported poor sleep quality across various specialities. Approximately similar results have been reported in earlier studies with higher rates among residents of clinical departments (24, 38, 39). Poor sleep quality among medical residents has been found to increase the risk of depressive symptoms, poor concentration, fatigue and daytime sleepiness (24, 39). Similarly, sleep deprivation, one of the components of sleep quality, has been found to induce stress and sympathetic activation, in turn increasing the risk for cardiovascular disorders (40). Moreover, poor sleep quality also produces oxidative stress, which enhances the risk for arterial injury and thus, can contribute to atherosclerosis, as was observed in this study (17, 41).

Previous studies have attempted to find an association between atherosclerosis and poor sleep quality among middle-aged patients having type 2 diabetes (42), middle-aged women (27), and middle age community-dwelling population (17). Present study expands to the previous study's findings by showing that poor sleep quality is associated with subclinical atherosclerosis among otherwise healthy young populations.

The present study has shown that factors that are traditionally considered as increasing the risk for atherosclerosis (dyslipidaemia, body mass index, smoking, fasting blood sugar, waist circumference) are less likely to play a dominant role in subclinical atherosclerosis at least in the presence of poor sleep quality (Table 2). Results of the present investigation are supported by a previous study by Domínguez et al. (43) who reported that short sleep duration (<6 h/day) and sleep fragmentation which are components of sleep quality (Figure 1) were associated with subclinical atherosclerosis even after controlling for other confounding variables e.g., age, gender, BMI, smoking, alcohol, physical activity, depression, perceived stress, blood pressure, risk for OSA, fasting glucose, total cholesterol and daily calorie intake. This research was furthered by Vallat et al. (44), who have shown that sleep fragmentation was associated with coronary artery calcification; however, this effect was mediated through increased neutrophil counts even after controlling for the age, gender, BMI, smoking status, blood pressure, use of antihypertensive medications, insomnia and obstructive sleep apnea. Cayres et al. (42) has also shown that truncal fat did not mediate the association between poor sleep quality and CIMT or femoral IMT (FIMT), supporting the results of the present study, where adiposity was measured using surrogate but validated markers of adiposity (body mass index, waist-hip ratio and waist circumference). Interestingly, a direct association between femoral IMT and poor sleep quality, but not with CIMT, has been reported in the past (42). It is possible that atherosclerosis starts in the femoral artery and then progresses to the carotid artery, perhaps due to greater sheer and tear in the femoral artery (42). Thus, previous studies also support the fact that perhaps, sleep quality is an important factor in the development of subclinical atherosclerosis. However, femoral IMT was not assessed in the present study and should be investigated in future.

Multiple studies have established the correlation between total sleep time and atherosclerosis risk factors (3–5). These studies have found a U shape association between total sleep time and increased CIMT (3–5). Though the meta-analysis has found an increased risk for diabetes mellitus, hypertension, coronary artery disease and obesity with total sleep time <6 h, short sleep was not associated with dyslipidaemia and depression (45). Another meta-analysis suggested that sleep duration <5 h/day was associated with metabolic syndrome, which is a risk factor for atherosclerosis (46). Similarly, an association between sleep disturbance, long sleep duration (>8 h/day) and increased systemic inflammation (increased C-reactive protein and IL-6) was reported in another meta-analysis but not with the short sleep duration (47). Present study also showed that sleep duration was not associated with increased CIMT while sleep disturbance, sleep onset latency and sleep efficiency were associated with CIMT.

However, several questions remain to be answered, e.g., Is there a causal relationship?; Are the results of the present study applicable to subjects having traditional risk factors for atherosclerosis, e.g., elderly, those with diabetes, those having metabolic syndrome or established cardiovascular disorders and heavy smokers?; does the relative contribution of various risk factors, including sleep quality and disturbance differ with regards to atherosclerosis?; is increased CIMT reversible with the improvement in sleep quality or sleep duration? Finding answers to these questions in well-designed prospective studies in the future can have a large impact on the health of resident doctors and that of the general population as components of sleep quality are modifiable factors.

Like any other scientific study, this study also suffered some methodological shortcomings. First, the data were cross-sectional, and hence only association could be established. Second, physical activity, perceived stress and the effect of irregular sleep-wake cycles were not measured. Since the participants were medical residents, it was assumed that they did not have a sedentary lifestyle. Third, smoking and dietary habits were measured as dichotomous variables, which may have a dose effect on atherosclerosis. Fourth, the effect of alcohol intake could not be assessed as participants were hesitant to respond. However, since all the participants were engaged in active work, chances of having alcohol dependence were negligible. Fifth, assessment of the interaction between sleep quality, sleep deprivation and shift work on CIMT after controlling for the confounding variables is a new area that should be addressed in future studies. Lastly, since the participants were medical doctors with adequate knowledge of risk factors for atherosclerosis, it was possible that they were trying to keep their lifestyle as healthy as possible, and hence, results “cannot be generalized”.

Strengths of the study included the inclusion of a younger sample without any other comorbidities, unlike previous studies (3–5, 17, 27). Secondly, optimal measures were taken to reduce bias, e.g., participants were selected using simple random sampling, most of the measures were self-reported, investigators indulged in anthropometric, and CIMT assessment was blinded to the status of sleep quality. Third, contrary to earlier studies, the present study assessed not only sleep duration as one of the components of sleep quality but also the qualitative assessment of “restful” “sleep”.

## Conclusion

Poor Sleep quality is significantly associated with increased carotid intima-media thickness. Focusing on Sleep quality, being a modifiable risk factor, may prevent or reverse accelerated atherosclerosis; however, it remains to be answered in future studies.

**Current Knowledge/ Study Rationale**: Several studies have shown that sleep duration has a **“**J**”** or **“**U**”** shape association with carotid intima-media thickness (CIMT), which is a marker for subclinical atherosclerosis. Contrary to sleep duration, sleep quality is a more inclusive concept. Despite this knowledge, literature examining the association between sleep quality and CIMT is scarce. A few reports have examined it but in the middle-aged population. This study was aimed to assess if self-reported sleep quality has any association with CIMT in otherwise healthy adults- resident doctors.

**What this study adds**: This study showed that poor subjective sleep quality, especially prolonged sleep onset latency, poor sleep efficiency and sleep disturbance were associated with greater CIMT among otherwise healthy young resident doctors.

## Data Availability

The original contributions presented in the study are included in the article/**Supplementary Material**, further inquiries can be directed to the corresponding author/s.
