# Supplementary material for: Poor quality sleep is associated with greater carotid intima media thickness among otherwise healthy resident doctors
Source: Front Epidemiol. 2023 Jan 11;2:1044111. doi: 10.3389/fepid.2022.1044111 (PMC10910945; doi:10.3389/fepid.2022.1044111)
Supplement: Supplementary file 1 [file Datasheet1.pdf]

Sequence #	Group 1
1	101
2	2
3	31
4	111
5	84
6	83
7	110
8	16
9	50
10	116
11	121
12	64
13	6
14	10
15	40
16	70
17	1
18	108
19	14
20	105
21	38
22	77
23	85
24	81
25	72
26	19
27	99
28	82
29	107
30	43
31	36
32	22
33	68
34	94
35	8
36	51
37	29
38	21
39	4
40	35
41	48
42	92
43	54
44	89
45	63
46	28
47	80
48	55

49	18
50	34
51	59
52	114
53	52
54	88
55	75
56	39
57	96
58	66
59	12
60	117
61	57
62	27
63	15
64	11
65	97
66	24
67	56
68	65
69	98
70	103
71	90
72	7
73	76
74	73
75	44
76	20
77	3
78	118
79	91
80	30
81	25
82	60
83	26
84	62
85	71
86	45
87	17
88	9
89	119
90	5
91	49
92	69
93	53
94	102
95	33
96	95
97	78

98	109
99	58
100	37
101	120
102	47
103	42
104	61
105	104
106	100
107	87
108	23
109	67
110	106
